# An Overview of Current Statistical Methods for Implementing Quality Tolerance Limits

**DOI:** 10.1007/s43441-023-00598-y

**Published:** 2023-12-26

**Authors:** Rakhi Kilaru, Sonia Amodio, Yasha Li, Christine Wells, Sharon Love, Yuling Zeng, Jingjing Ye, Monika Jelizarow, Abhinav Balakumar, Maciej Fronc, Anne Sofie Osterdal, Tim Rolfe, Susan Talbot

**Affiliations:** 1 PPD, Part of Thermo Fisher Scientific, 929 North Front Street, Wilmington, NC 28401-3331 USA; 2 Biometrics, Medical and Nutritional Science, Danone Nutricia Research, Utrecht, The Netherlands; 3 Central Statistical Monitoring (CSM), Data Science and Digital Innovations (DSDI), Global Statistical and Data Sciences (GSDS), BeiGene, Wuhan, China; 4 Roche Products Ltd, 6 Falcon Way, Shire Park Welwyn, Garden City, AL7 1TW UK; 5 MRC Clinical Trials Unit at UCL, Institute of Clinical Trials and Methodology, London, UK; 6 Data Science and Digital Innovations (DSDI), Global Statistical and Data Sciences (GSDS), BeiGene, Fulton, MD USA; 7 Center of Excellence for Statistical Innovation (CESI), Statistical Sciences & Innovation (SSI), UCB BIOSCIENCES GmbH, Alfred-Nobel-Strasse 10, 40789 Monheim, Germany; 8 Health Data Insights and Design, Global Clinical Operations, Novartis Healthcare Pvt. Ltd, Hyderabad, India; 9 Central Monitoring and Data Analytics, Global Clinical Operations, GSK, Warsaw, Poland; 10 SGH Warsaw School of Economics, Warsaw, Poland; 11 ALK, Bøge Alle 1, Hørsholm, Denmark; 12 Central Monitoring and Data Analytics, Global Clinical Operations, GSK, London, UK; 13 Amgen Inc, Thousand Oaks, CA USA

**Keywords:** Centralized statistical monitoring, Quality tolerance limits, Risk-based monitoring, Data quality, Risk identification, Quality by design

## Abstract

**Background:**

In 2016, the International Council for Harmonisation of Technical Requirements for Pharmaceuticals for Human Use updated its efficacy guideline for good clinical practice and introduced predefined quality tolerance limits (QTLs) as a quality control in clinical trials. QTLs are complementary to Quality by Design (QbD) principles (ICH-E8) and are one of the components of the risk-based clinical trial quality management system.

**Methods:**

Currently the framework for QTLs process is well established, extensively describing the operational aspects of Defining, Monitoring and Reporting, but a single source of commonly used methods to establish QTLs and secondary limits is lacking. This paper will primarily focus on closing this gap and include applications of statistical process control and Bayesian methods on commonly used study level quality parameters such as premature treatment discontinuation, study discontinuation and significant protocol deviations as examples.

**Conclusions:**

Application of quality tolerance limits to parameters that correspond to critical to quality factors help identify systematic errors. Some situations pose special challenges to implementing QTLs and not all methods are optimal in every scenario. Early warning signals, in addition to QTL, are necessary to trigger actions to further minimize the possibility of an end-of-study excursion.

## Background

**Figure 1:**
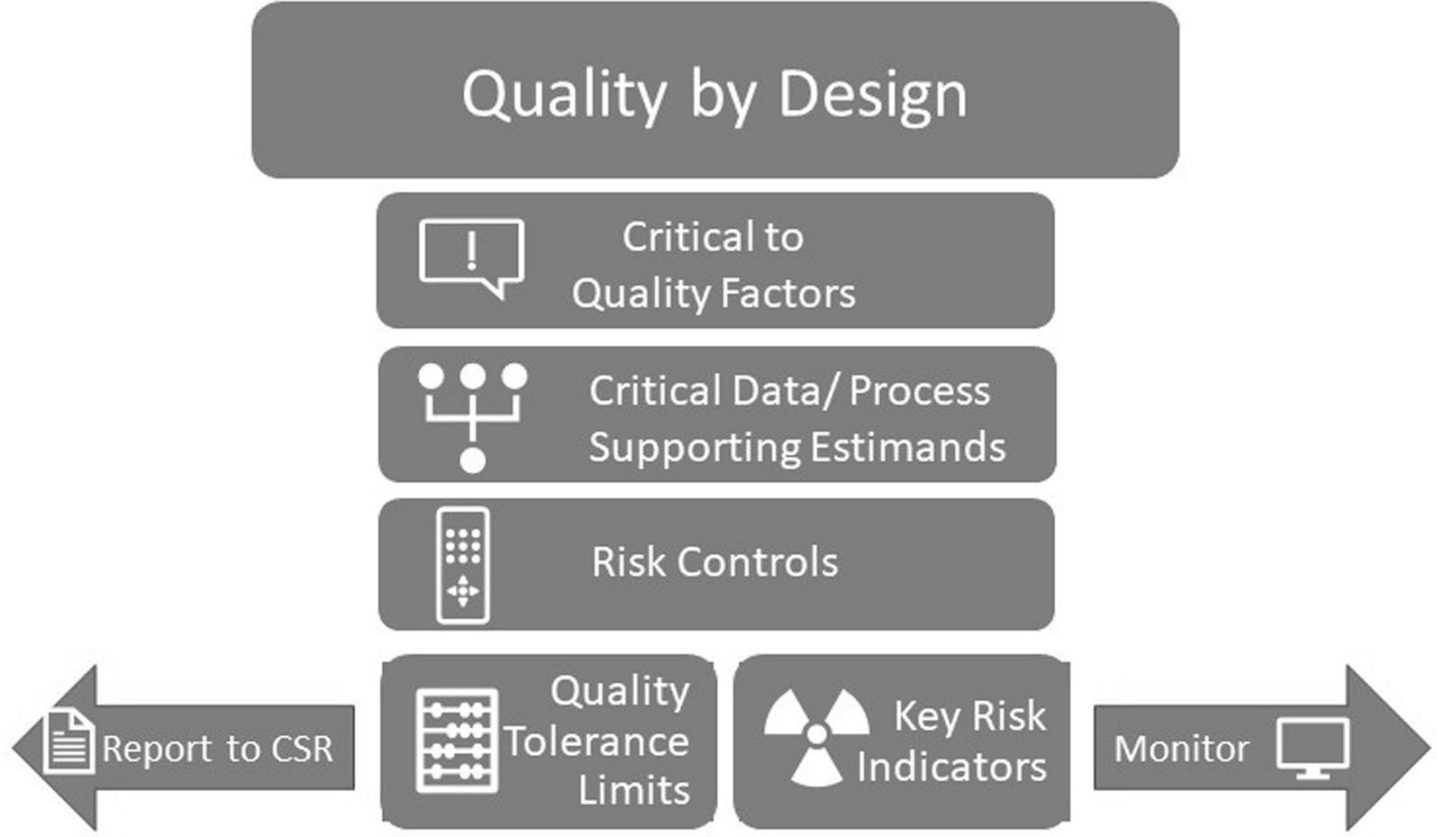
Risk-Based Quality Management (RBQM) components.

According to ICH E6 R2 [1], the main objective of predefined quality tolerance limits (QTLs) is to establish a proactive process to detect systematic issues that can impact participant safety or the reliability of the trial results. The QTL process is implemented at trial level focusing on Critical to Quality factors (CtQ) that impact trial quality. According to TransCelerate, the establishment of QTLs is complementary to Quality by Design (QbD) principles (ICH-E8) and is one of the components of the risk-based clinical trial quality management system (Fig. 1). The Clinical Trials Transformation Initiative (CTTI) Quality by Design Project—Critical to Quality Factors Principles Document [2] emphasizes the importance of QbD by providing guidance on CtQ factors to consider when designing a protocol. Those are most likely to correlate with critical processes and data that are essential to ensure participant protection and the reliability of trial results.

QTL is a level, threshold or value that defines an acceptable range which is associated with a parameter that is critical to quality. A deviation from a threshold, also called an “excursion”, during the conduct of the trial may indicate a systematic issue that could impact participants’ safety or reliability of trial results. TransCelerate has published guidance in “Risk-Based Quality Management: Quality Tolerance Limits and Risk Reporting,” [3] which includes references to the use of statistical process control (SPC) methods to construct QTLs. In SPC as further described below, a process is “in control” if, over time, the parameter varies due to random noise only. The general aim of QTLs is to set upper limit (UL) and lower limit (LL) so that the probability of an excursion is low for an in-control process (i.e., false alarm probability) and high for an out-of-control process (i.e., sensitivity or power).

QTLs are set-up with the following template fields taken from the TransCelerate RBQM and Risk Reporting Appendix [3]:

1. Parameter: A critical to quality parameter that is going to be monitored.
2. Definition: How will the QTL be measured and calculated.
3. Justification for the Parameter.
4. Unit of Measure: e.g., number, proportion.
5. Expected Values: What is the expected value based on historical data and expert opinion.
6. Justification for Expected Values.
7. Quality Tolerance Limit: What is the limit.
8. Justification for the Quality Tolerance Limit: include methodology.
9. Planned Mitigation Actions: What will be done if there is a risk of crossing the QTL or if it is crossed.

The selection of methods will depend on the QTL and the data being measured such as rates, proportions, or time to event. Justification for the QTL will include the reason for selecting the method to construct limits and false alarm probability (type 1 error) used. Key Risk Indicators (KRIs) are often used with QTLs to help control risks. KRIs and QTLs differ in that KRIs are typically measured at the site level to inform site monitoring activities, while QTLs are a higher-level indication of overall quality in a trial. Specific methodologies used to construct tolerance limits are discussed in detail in the sections below.

QTLs may be one-sided as that allows the prespecified false alarm probability (*α* or type 1 error) to be allocated to the side of interest.

ICH guidelines [1, 4] (ICH E6 R2 and E8 R1) state that historical data should be used to set QTLs, but obtaining such data can be challenging especially for new/novel disease areas, rare diseases, etc. The framework for establishing QTLs in clinical trials has been discussed elsewhere [5–7] proposing alternatives for historical data [8, 9] including clinical and safety science knowledge or meta-analysis of data from external sources such as TransCelerate Placebo Data sets, FDA and Clinicaltrials.gov websites and publications.

Examples of methods being utilized by members of the PSI/EFSPI/ASA-BIOP CSM and QTL Special Interest Group include Beta-Binomial models, Bayesian hierarchical models, Observed–Expected (O–E) control charts, Observed/Expected (O/E) control charts; Cumulative proportion charts, CUSUM plots; standard deviations, rates, and observation counts.

## Commonly Used Methods

If a clinical trial is to meet regulatory requirements, it should be conducted through processes that are stable and repeatable. More precisely, the processes must be capable of operating with little variability around the target of trial quality characteristics. Statistical process control (SPC) is a powerful collection of problem-solving tools useful in achieving process stability and improving trial quality through reduction of variability [10]. In any process, regardless of how well designed or carefully maintained it is, a certain amount of inherent or natural variability will always exist. This natural variability or random error is the cumulative effect of many small un-avoidable causes. In the framework of statistical quality control, this natural variability is often called “stable system of chance causes”. A process that is operating with only chance causes of variation or random errors present is said to be in-control. Other kinds of variability may occasionally be present in the output of a process. This variability in key quality characteristics of a trial process can arise from improper trial conduct, not adhering with protocol or other causes creating systematic irregularities. Such variability is large when compared to random errors and it usually represents an unacceptable level of process performance. We refer to these sources of variation that are not random as “assignable causes”. A process that is operating in the presence of assignable causes is said to be out-of-control.

A major objective of statistical process control is to quickly detect the occurrence of assignable cause of process shifts so corrective action may be taken before end-of-study quality breach. Among the major tools in SPC, control charts are widely used.

As opposed to frequentist methods, which consider the quantity of interest to be fixed but unknown, Bayesian methods consider the quantity of interest to be a random variable with a distribution that reflects the current belief or knowledge about that quantity. As such, under the Bayesian paradigm our pre-trial evidence about a QTL parameter, *e.g., ‘true proportion of participants randomized who prematurely discontinue treatment’* can be summarized by a distribution reflecting our uncertainty. This pre-trial evidence can be used in various ways, for example to derive the distribution of the data we expect to observe in the future, based on current evidence, or it can be combined with on-trial evidence into post-trial evidence in a mathematical fashion based on Bayes’ theorem. Bayesian methods are therefore appealing in the context of QTLs where incorporation of historical, i.e., pre-trial information, is considered [11].

Two examples of Bayesian methods namely, Beta-Binomial model and Bayesian Hierarchical Model currently used in practice for QTL monitoring are described below. Formal evaluation of these methods’ operating characteristics is beyond the scope of this paper.

## Statistical Process Control: Control Charts

Due to control charts being widely used and considered one of the more technical statistical process control approaches, greater focus has been placed on using control charts in implementing QTLs and monitoring CtQ factors.

A control chart is a graph with control boundaries, and it is used to analyze and judge whether a process is in-control. There are 3 commonly used control charts used to implement quality tolerance limits namely O–E Difference, O/E Ratio, and cumulative proportion. QTLs are generally implemented as control boundaries or control limits. However, QTLs can be implemented with QTL being fixed alongside control limits also called secondary limits to provide early signals of risk. The latter is the approach described in the sections below where control limits and secondary limits are used interchangeably.

## Observed Minus Expected Difference Chart (O–E Difference)

The O-E difference chart [12] can be applied to the cumulative sum of differences between observed events and expected events The X-axis of the O-E chart can be for example the cumulative number of participants; the cumulative number of participants visits or the cumulative exposure time of treatment. The Y-axis is the number of events of interest that occurred minus the expected value.

O–E chart is appropriate when the total number of participants is known in advance before clinical trial commencement, when the event of interest for each participant is binary (i.e., Success/Failure, Yes/No), and when the expected probability of the event of interest is constant for each participant in line with participants enrolled in clinical trial sharing similar baseline characteristics following the same protocol. QTLs are set based on expectations from historical data or expert knowledge.

The contribution of each participant to the total number of events of interest is identical and therefore, for each participant, the occurrence of the event can be regarded as a Bernoulli event. Moreover, since participants are independent of each other, the theoretical distribution of the occurrence of the event in a trial is Binomial. At each monitoring occasion, if the process is experiencing more events than expected the line will jump upward, whilst if no event occurs, the line will trend downward (since expected events continue to accumulate). A process in line with expectations will have a stable line with natural fluctuations around zero. When calculating quality tolerance limit or secondary limit, one can use exact control intervals based on the binomial distribution or their asymptotic counterparts.

**Figure 2:**
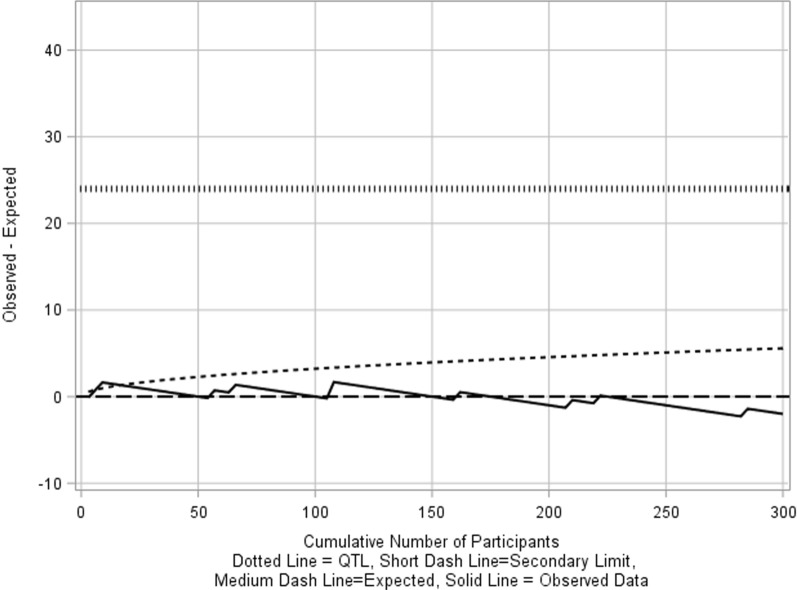
O–E chart of premature treatment discontinuation.

For example, a phase III clinical trial plans to enroll 300 participants, and one of the target QTL parameters for the trial is the proportion of participants who discontinue the study drug prematurely. The expected value based on historical data shows an average discontinuation rate of 4% and it is decided that the parameter’s QTL is set at 12% the maximum (fixed) limit throughout the conduct of trial which corresponds to a difference of 24 events. An O–E chart is shown in Fig. 2. Only the upper limits are plotted because an excess number of events are of concern.

The secondary limits are set corresponding to the upper 95th quantile (1-sided *α* = 0.05) of a normal approximation to the binomial distribution (henceforth termed binomial-asymptotic method) with n cumulative number of observations minus the expected value. This example uses an in-control process where the actual discontinuation rate and the expected rate are the same. The binomial-asymptotic method assumes that the sample size is large enough, otherwise, for smaller sample size trials, exact control limits are considered more appropriate to achieve tradeoff between sensitivity and specificity.

**Figure 3:**
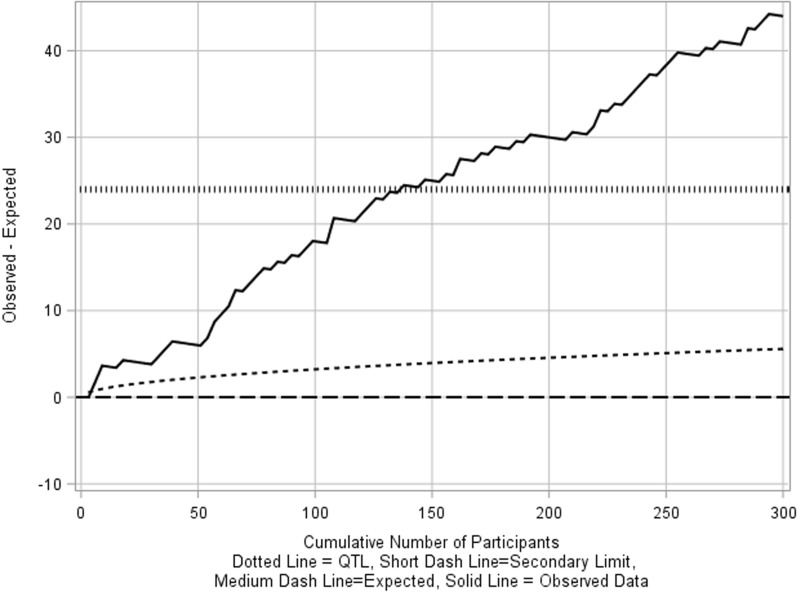
O–E chart showing early crossing of secondary limits when the actual proportion of event is higher than expected.

If the actual probability of early termination per participant is 0.2 (much greater than 0.12), without immediate actions in place, as the rate of participants enrollment increases, the process can spiral out-of-control (Fig. 3).

## Observed/Expected Chart (O/E Ratio)

O/E chart [12] can be applied to the ratio of the observed value to the expected value. The X-axis of the O/E chart is usually the cumulative count of units in the trial, such as the cumulative number of enrolled participants or cumulative study or treatment exposure. The Y-axis corresponds to the ratio of the number of events of interest that occurred to the expected value.

**Figure 4:**
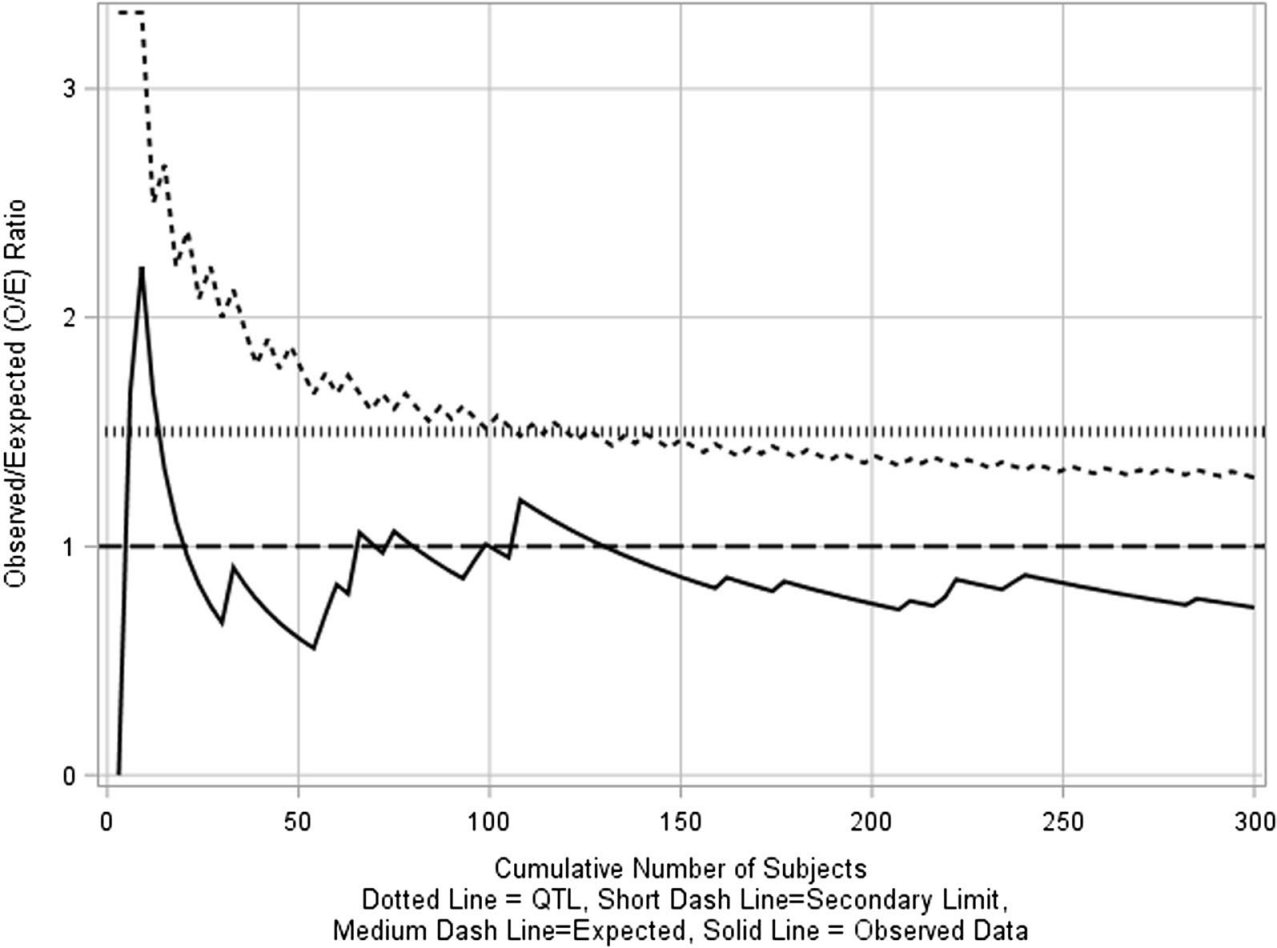
O/E chart on significant protocol deviations.

An example is significant protocol deviations. A Poisson distribution can be used to calculate the control limit [12] as the enrolled participants are independent of each other and the focus for the clinical study team is the cumulative count of significant deviations that accumulate over time. Consider an example clinical trial with a planned sample size of 300 and 0.1 expected significant deviation count per participant or a cumulative total of 30 significant deviations among 300 participants with a tolerance limit set at 45 significant deviations. An O/E chart can be constructed with the tolerance limit for O/E ratio fixed at 1.5 (i.e., 45/30) and the secondary limits are calculated using 95th quantile (1-sided *α* = 0.05) of the Poisson distribution (Fig. 4). This example uses an in-control process where actual protocol deviation rate in the trial is equal to the expected deviation rate.

As seen in Fig. 4, the secondary limits exceed QTL during the earlier period in the study and based on the fixed QTL, an out-of-control process is detected initially which subsequently returns into control with increasing number of participants. Hence, it may be more appropriate to monitor parameters using O/E charts after the number of participants exceed 30.

## Cumulative Proportion Chart

The cumulative proportion chart is applied to a cumulative or rolling proportion of events (ignoring exposure) that are expected to be constant throughout a trial, whether the final size is known or not. Considering the example of premature treatment discontinuations due to reasons unrelated to safety or pharmacologic outcomes, several methods have been used to construct control limits as secondary limits or QTLs for a cumulative proportion with similar assumptions indicated above for O–E with the contribution of each participant to the total number of events of interest being identical and hence the occurrence of the event can be regarded as a Bernoulli event. As seen in the O-E Chart or the O/E chart, the control limits can be considered secondary limits with a maximum fixed limit specified as the QTL.

### Secondary Limits Using the Binomial Quantile method

The upper and lower control limits can be determined using the 5th and 95th quantiles (2-sided false alarm probability, *α* = 0.1) of the underlying binomial distribution using the pre-specified expected (historical) proportion and the observed sample size at that point. The binomial quantiles [13] indicate that if the ongoing data follow the expected binomial distribution, the probability of false alarm should be controlled at $1-P(LCL<X<UCL)=\alpha$.

### Binomial Exact Secondary Limits

To set-up secondary control limits based on an exact confidence interval, the estimated probability of event for the ongoing study is set to the historical rate or proportion assuming they are the same. The exact control limits are based on the relationship between the binomial and F families of distributions [14, 15].

### Asymptotic Secondary Limits

Asymptotic secondary control limits, based on asymptotic normal distribution are calculated under the assumption that the cumulative proportion for the study is known and equal to the historical proportion.

The binomial quantile and asymptotic methods tend to produce an upper limit lower than that from the binomial exact method earlier in the trial resulting in potentially more breaches that will need investigation. Cumulative proportion charts don’t account for treatment or study exposure. Quantile and asymptotic control limits rely on large sample size. Hence, it may be more appropriate to monitor parameters using cumulative proportion charts after the number of participants exceeds 30.

**Figure 5:**
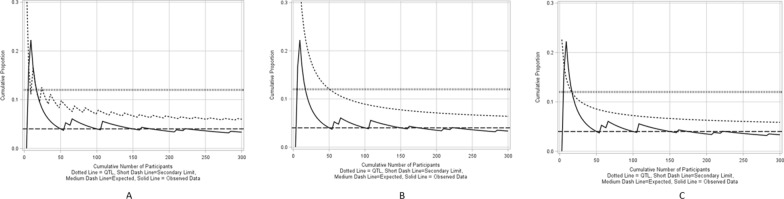
**A** Cumulative proportion chart using a binomial quantile method for an in-control process, **B** Cumulative proportion chart using an exact method for an in-control process, **C** Cumulative proportion chart using asymptotic method for an in-control process.

Figure 5A–C show a cumulative proportion chart on monitoring premature treatment discontinuation for an example clinical trial with a sample size of 300 using Quantile, Exact and Asymptotic methods for a process that is in-control. The historical expectation is set at 0.04 with the actual premature treatment discontinuation rate being equal to the expectation.

**Figure 6:**
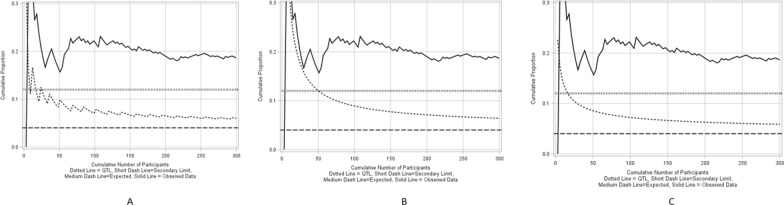
A Cumulative proportion chart using a binomial quantile method for an out-of-control process, **B** Cumulative proportion chart using exact method for an out-of-control process, **C** Cumulative proportion chart using asymptotic method for an out-of-control process.

Figure 6A–C show a cumulative proportion chart on monitoring premature treatment discontinuation for the same example clinical trial using Binomial Quantile, Exact and Asymptotic methods for a process that is out-of-control where the actual premature treatment discontinuation rate is 0.2, which is much larger than the expectation. The maximum quality tolerance limit is set at 0.12.

## Beta-Binomial Model

For events of binary type, the total number of events observed in the trial follows $B(n,p)$ distribution, where $n$ is the number of the samples and $p$ is an unknown parameter. From the Bayes point of view, the historical data can be used for the prior distribution of the unknown parameter $p$. Since the total number of an event follows the binomial distribution, a beta distribution is used as the prior distribution of $p$.

The prior information on $p$ from ${n}_{0}$ Bernoulli events can be denoted by a Beta distribution whose mean is ${P}_{0}$, i.e., $Beta({n}_{0}{p}_{0},{n}_{0}{(1-p}_{0}))$. If ${n}_{c}$ future Bernoulli events are observed and ${T}_{c}$ of them are success events, letting ${p}_{1}=({n}_{0}{p}_{0}+{T}_{c})/{n}_{1}$ and ${n}_{1}={n}_{0}+{n}_{c}$, according to the likelihood of the observed data, the posterior distribution of p is also beta distribution [16, 17]: $Beta({n}_{1}{p}_{1},{n}_{1}{(1-p}_{1}))$.

Based on this, the control limits for a future sample of $n$ Bernoulli events with $T$ successes can be deduced. Given $n$ and $p$, the distribution of $T$ is binomial, and the posterior predictive distribution of $T$ can be derived as a beta-binomial distribution. That is, for future samples $D=\left\{{x}_{i}\right\},i=1,..,n$, the posterior predictive distribution of $T ({\sum }_{i}{x}_{i})$ can be expressed as:

1 $$f\left( {T|D,T_{c} } \right) = {{C_{n}^{T} \left( {B\left( {T + n_{1} p_{1} ,n - T + n_{1} \left( {1 - p_{1} } \right)} \right)} \right)} \mathord{\left/ {\vphantom {{C_{n}^{T} \left( {B\left( {T + n_{1} p_{1} ,n - T + n_{1} \left( {1 - p_{1} } \right)} \right)} \right)} {\left( {B\left( {n_{1} p_{1} ,n_{1} \left( {1 - p_{1} } \right)} \right)} \right),\,0 \le T \le n}}} \right. \kern-0pt} {\left( {B\left( {n_{1} p_{1} ,n_{1} \left( {1 - p_{1} } \right)} \right)} \right),\,0 \le T \le n}}$$

The median of the posterior predictive distribution of $T$ can be calculated easily through the derivation above. The median is used as a measurement to assess whether the current observation exceeds the QTL threshold, provided that the general performance of current data can be expressed by the median of the posterior predictive distribution.

Using an example of proportion of participants with protocol deviations (PDs) of special interest in a trial, historical data shows that the average proportion and 95th quantile of participants with PDs of interest are 17.95% and 27.17%, respectively. The prior distribution of $p$ based on historical data was deduced as $Beta\left(\mathrm{13.6,58.5}\right)$.

**Figure 7:**
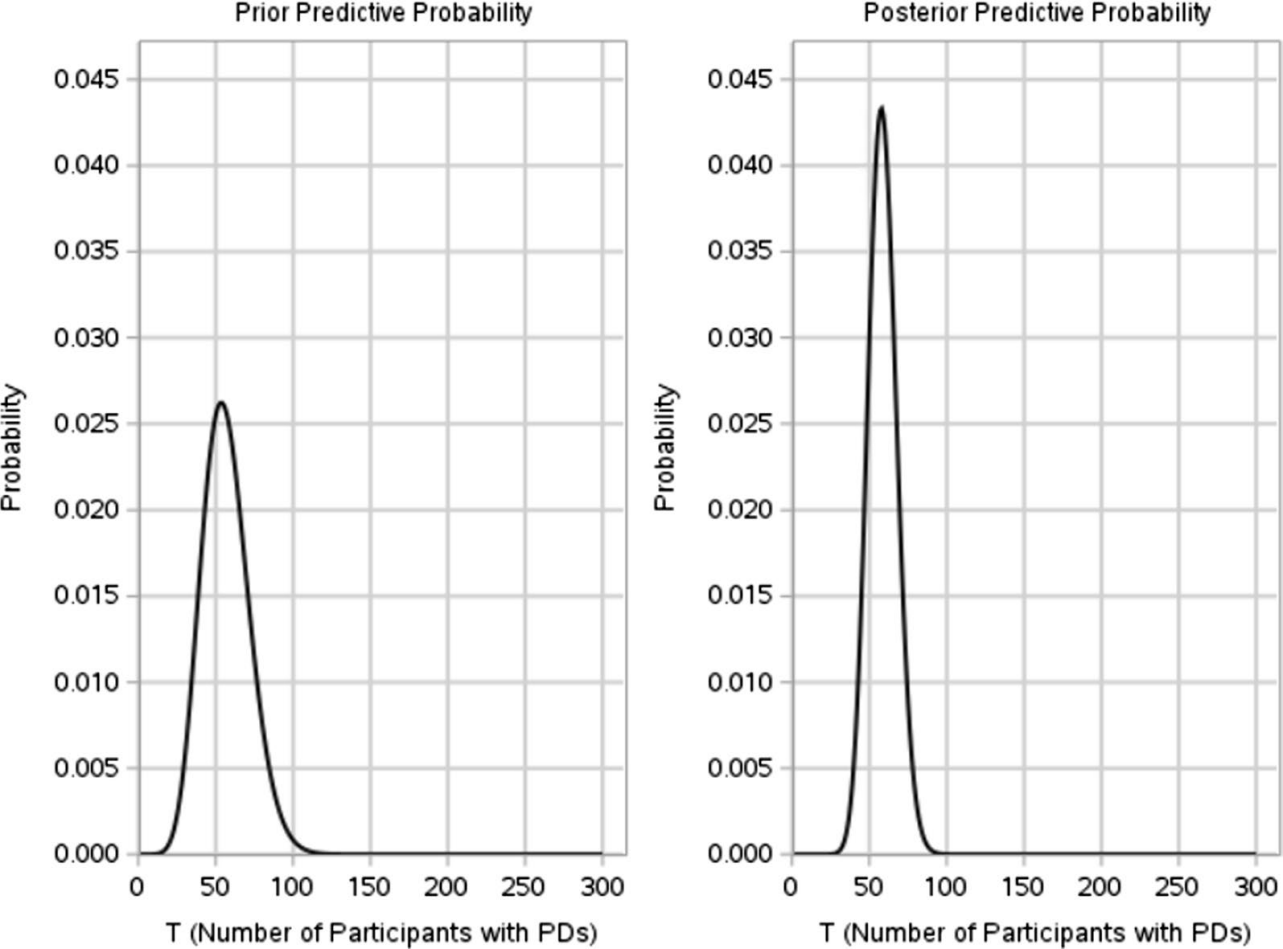
Predictive density of *T*.

Assuming that this QTL parameter was monitored in a trial with 300 enrolled participants and the expected proportion of participants with PDs is 17.95% (i.e., generating a sample of size 300 from the Bernoulli (0.1795)), based on the above derivation, the prior and posterior predictive probability density diagram of $T$ (number of participants with PDs) in this process can be obtained, as shown in Fig. 7.

**Figure 8:**
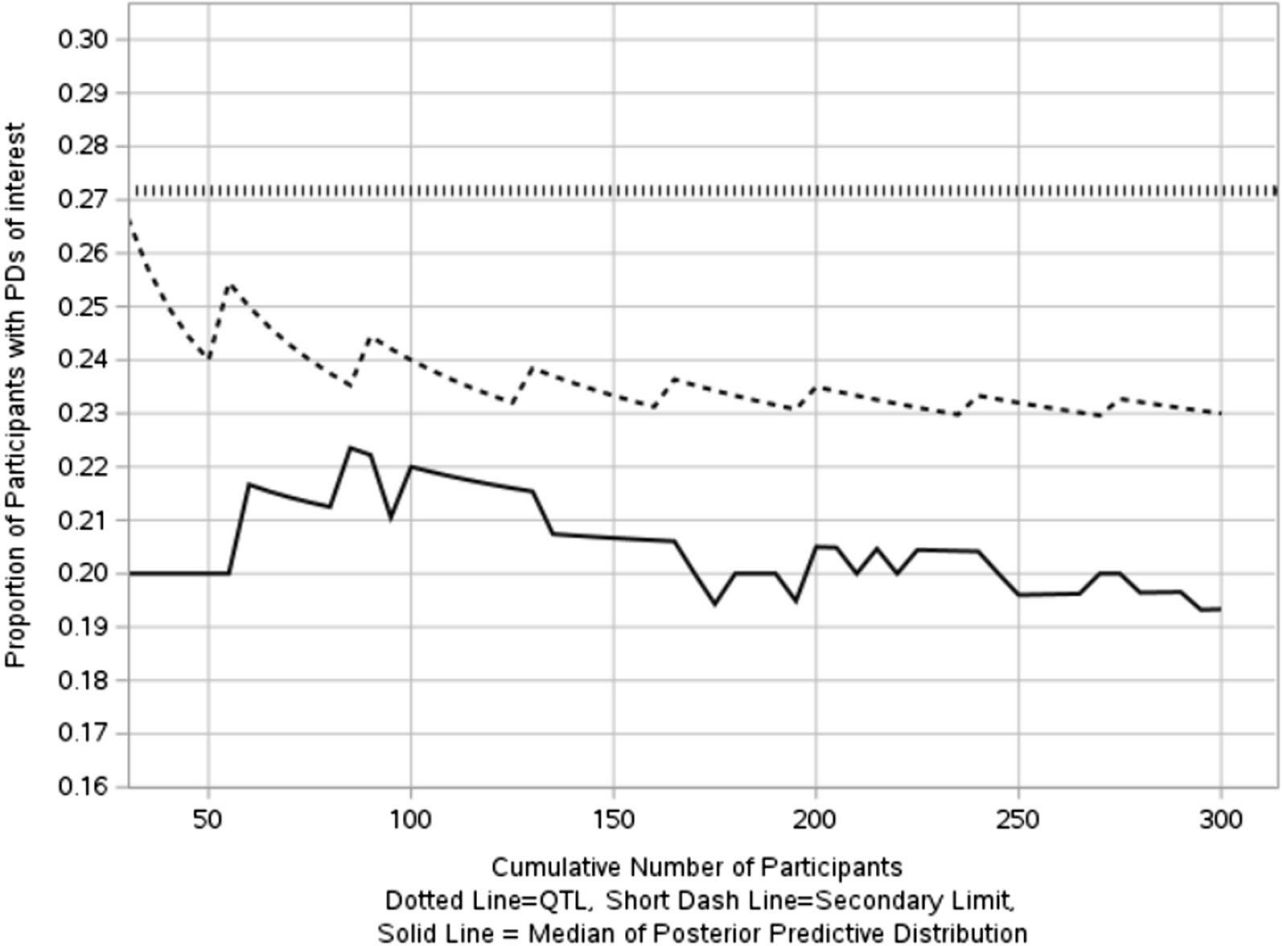
In-control process for proportion of participants with PDs of interest.

The quality tolerance limit uses the 95th quantile (27.17%) of historical data with the 80th quantile of the prior predictive distribution specified as the secondary limit (rendering sufficient time for actions) in the QTL monitoring process. The control chart using median of the posterior prediction distribution as the measurement to assess future data are shown in Fig. 8.

As previously mentioned, monitoring QTL parameters starts when the sample size is large enough, in this example, monitoring begins when the number of enrolled participants is 30, hence the above figure only shows the results when the number of participants ≥ 30. As can be seen from Fig. 8, the median of the posterior distribution doesn’t breach QTL threshold, which indicates an in-control process.

**Figure 9:**
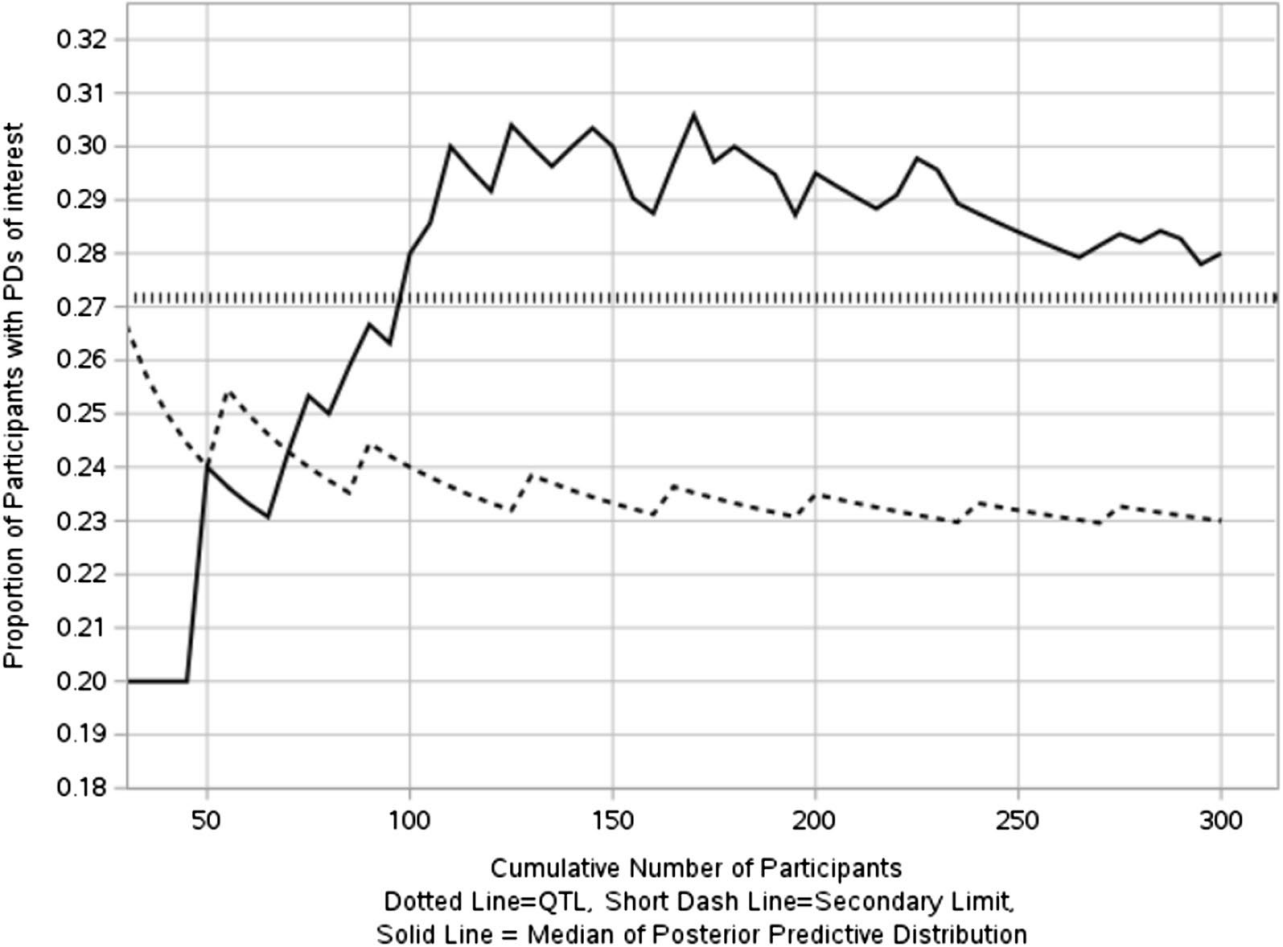
Out-of-control process for proportion of participants with PDs of interest.

Figure 9 shows an out-of-control process on monitoring proportion of participants with interested PDs for the example of Bernoulli (0.30) event, the median of posterior predictive distribution of PD proportion breach both tolerance limits successively.

This method is generally applicable to large sample size trials. Moreover, since the trial-specific assessment (median from the posterior predictive distribution) uses both historical and current data with thresholds established using historical data, it is important that the historical data are sufficiently homogeneous and similar in indication and ideally with a similar compound under investigation.

In practice, differences between trial data and historical data may be found as clinical trials are not like manufacturing. For example, there may be none, or insufficient historical data available for new clinical trials for new compounds or new indications or rare diseases. Should the historical data be inconsistent with current data, there is a potential for an inflated false alarm probability and may cause excursions on each run. One can use the minimal sufficient statistic method by Evans and Moshonov[18] to check for prior-data conflict.

When prior-data conflicts exist, one can consider adjusting the threshold or using power priors to update the posterior predictive distribution to prolong the run length of the process [19].

## Bayesian Hierarchical Model

This method is based on a Bayesian meta-analysis of clinical trials data presented by Berry et al. [20]. Technically, the QTL is defined in terms of the quantiles of the posterior distribution of the metric of interest $M$ but understanding at this level is not required to interpret its output.

Suppose we have $n$ independent observations of a metric $m$, $d = \{{m}_{1}, {m}_{2}, \dots , {m}_{n}\}$. We assume that $d$ is a random sample from a population with distribution $f$, which is itself random and defined by a set of parameters *θ*. The value of θ is unknown, so we define a prior distribution $\pi$(*θ* | *ψ*), where *ψ* is a set of hyper parameters with distribution h(*ψ*). This is a standard hierarchical Bayes model, so that,

2 $$p\left( d \right) = \frac{{p\left( {d|\theta } \right)}}{p\left( d \right)} = \frac{{\int {f\left( \theta \right)} \,\pi \left( \psi \right)\,h\left( \psi \right)\,d\psi }}{{\iint {f\left( \delta \right)}\,\pi \left( \psi \right)\,h\left( \psi \right)\,d\theta d\psi }}$$

For binary data, we suppose ${r}_{i}$ of ${k}_{i}$ participants at site $i$ experience at least one event, $i=1,\dots , n$ and that probability that a particular participant experiences the event is independent of whether any other participant does so. Clearly, ${R}_{i} \sim Bin({p}_{i}, {k}_{i})$, so here $\theta ^{\prime} = \left\{ {p_{1} , \ldots ,p_{n} } \right\}$, where ${p}_{i} = {r}_{i}/{k}_{i}$. We set *π* (*θ* | *ψ*) ~ $Beta\left(a, b\right),$ so *ψ*’ = {a b}. h(.) ~ U(0, 10) as used by Berry et al. which is an empirically reasonable choice of hyperprior for both a and b, though other choices are perfectly possible. The rbqmR package that performs this analysis can be found at https://github.com/openpharma/rbqmR.

Whether or not reference information is available for $m$, QTLs are based on the quantiles of the posterior distribution of $p\left(d\right)$. In the simplest case, when reference information is not available, the quantiles themselves can be used as the QTLs.

For example, suppose that $\{{m}_{L}, {m}_{U}\}$ is a $(1-\alpha )\%$ posterior credible interval for M. It does not matter whether the credible interval is one or two-sided. We calculate the proportion of sites ${p}^{*}$ whose observed metric m_i_ lies outside the interval and compare this figure to a pre-specified multiple of $\alpha$, ${\alpha }^{*}= Z\alpha$ for some $Z > 0$. If ${p}^{*}>{ \alpha }^{*}$ the QTL has been exceeded. For example, in a study of 20 sites, we might set $Z$ to 2 and α to 0.1. Then if 5 or more sites have a value of m_i_ that lies outside the range $\{{m}_{L}, {m}_{U}\}$, the QTL has been exceeded, since 20 × 0.1 × 2 = 4.

The side of the interval can depend on the metric being monitored. For example, on the assumption that the ideal rate of baseline protocol deviations is 0, then a one-sided lower credible interval would be appropriate. On the other hand, if a treatment has known incidence of adverse events, then a two-sided interval would be appropriate for monitoring the proportion of participants reporting AEs associated with the treatment.

If some knowledge of the expected range of the metric is known, either from reference data or by other means, the QTLs can be based on some summary of the posterior distribution of the metric. Examples include:

- The mean posterior probability of a baseline protocol deviation should be less than 0.05.
- The chance of a treatment-related SAE should lie in the range [3%, 8%] with probability greater than 0.6.

The use of a Bayesian hierarchical model removes the need for reference data and allows each study to act as its own control. This both reduces the burden of RBQM on study teams and reduces the risk that QTLs are inappropriately regarded as targets.

A fully worked example of this methodology can be found along with the required code in the rbqmR package on GitHub open pharma.

## Discussion

This paper described a range of statistical methods to establish control limits as QTLs, secondary limits or both, consistent with the objectives outlined in ICH E6 R2. In addition, these methods can be implemented for early warning systems, to further minimize the possibility of end-of-study breach on quality. This paper summarizes currently available methods but is not exhaustive. For example, there are many other types of control chart available depending on the outcome parameter, such as X-bar- R chart (continuous), p-chart (proportion or count) and np-charts (attribute as nonconforming or conforming), etc.

The methods outlined have their pros and cons. In general, O-E difference, O/E ratio and cumulative proportion control charts that use binomial quantile or asymptotic methods are effective in detecting an out-of-control process especially if the expectations are well-defined, justified and the trial sample size is large enough. The general rule of thumb is to implement these methods in trials with a sample size of 50 participants or more. The Binomial exact control limits can somewhat overcome the sample size limitations and can be run when sample size is small.

One of the key advantages of using the Beta-Binomial model is its non-reliance on well-defined expectations as required by control charts. However, the methodology relies on use of similar historical studies to generate the prior predictive probability distribution and tends to perform better after accrual of sufficient participants. Inconsistencies between current study and historical studies can create prior-data conflict and render the approach less desirable.

The key advantages of the Bayesian Hierarchical Model are that it takes account of possible variability between sites and, by defining action and warning limits in terms of the posterior, also allows a study to act as its own control. This both reduces the workload on study teams by removing the need to find and summarize relevant historical data and reduces the risk of false alarm caused by inaccurate expectation.

O–E and cumulative proportion charts and beta-binomial methods are applied to proportions. The O/E ratio chart can be extended to rates accounting for exposure.

A general rule of thumb advocates running the approaches described in this paper for the first monitoring review after 30 participants to minimize risk of false alarm.

Formal evaluation of the methods, including best practices will be the subject of a subsequent paper.

Small, complex designs, phase 1 studies, or platform studies, or Basket Designs or early parts of a seamless design may not be appropriate for complex statistical methods of early warning system using QTLs if the trial set-up, complexity, or duration does not allow for a close follow-up of risk. Many robust statistical approaches may not be appropriate in small trials, and it should be acknowledged that not all situations can benefit from implementing QTLs via complex analytics and careful consideration needs to be given to identify the optimal methodology for the scenario. For example, for some smaller trials a simple count of events may be sufficient to establish whether a QTL excursion has occurred, e.g., no more than 2 participants with missing pharmacokinetics (PK) assessments in a 20-participants Phase 1 cohort.

Multiplicity should be considered depending on the number of parameters and frequency which they are monitored, but it is important to consider this within the context of RBQM. In general, the goal of QTLs is to trigger an investigation into the root cause and potential mitigation if issues are observed for parameters critical to the success of a study, so the impact of a false positive is of far less concern than it would be for an assessment of treatment efficacy. However, longitudinal QTL assessment may result in many correlated analyses throughout the course of a study targeting the same pre-specified variable/parameter as a QTL target, so an appropriate False Discovery Rate correction could be considered. Additionally, an excursion on one QTL target does not preclude investigating other targets (regardless of whether they encountered excursions). Like situations in which several safety analyses are performed, where typically multiplicity adjustment is not of concern and overall safety profile is deemed more important, a breach encountered through QTLs is relative to all pertinent risks which determine the best course of corrective action.

Although two-sided limits are recommended in most applications for quality control, one-sided control limits may be more appropriate in clinical trials so that the prespecified false alarm probability (*α* or type 1 error) can be allotted to the side of interest (lower or upper), which would provide greater power for identifying noteworthy signals in the direction of interest. The decision to use one-sided or two-sided control limits is generally based on the variable of interest and whether there is a strong indication to focus on signals in a single direction.

Selection of appropriate denominators for proportions requires consideration. Dependent upon the QTL parameter an adjustment may be necessary for study or treatment exposure (through annualized events over time) to ensure appropriate weight is given to each observation.

ICH E6 (R2) requires that QTLs be prespecified before a protocol is implemented. When there is a significant difference between current data and historical data, QTL parameters will encounter an excursion based on threshold determined by historical data at the early stage of a trial. With diligent application of RBQM principles, QTLs can help detect, at the earliest possible opportunity, potential issues in the critical data being captured. Early detection of issues should allow sufficient time for study teams to implement mitigation activities which ultimately result in the issue being resolved prior to the end of a study. This in turn results in clinical trials which maximize the chance of providing clear and robust interpretation of the trial’s primary objectives.

In conclusion, application of quality tolerance limits to parameters that are associated with critical to quality factors help identify systematic errors that compromise study integrity. A suite of tools has been described, to aid with the implementation of QTLs using appropriate statistical methodology.
