# Correction: An Overview of Current Statistical Methods for Implementing Quality Tolerance Limits

**DOI:** 10.1007/s43441-024-00621-w

**Published:** 2024-01-20

**Authors:** Rakhi Kilaru, Sonia Amodio, Yasha Li, Christine Wells, Sharon Love, Yuling Zeng, Jingjing Ye, Monika Jelizarow, Abhinav Balakumar, Maciej Fronc, Anne Sofie Osterdal, Tim Rolfe, Susan Talbot

**Affiliations:** 1 PPD, Part of Thermo Fisher Scientific, 929 North Front Street, Wilmington, NC 28401-3331 USA; 2 Biometrics, Medical and Nutritional Science, Danone Nutricia Research, Utrecht, The Netherlands; 3 Central Statistical Monitoring (CSM), Data Science and Digital Innovations (DSDI), Global Statistical and Data Sciences (GSDS), BeiGene, Wuhan, China; 4 Roche Products Ltd, 6 Falcon Way, Shire Park Welwyn, Garden City, AL7 1TW UK; 5 MRC Clinical Trials Unit at UCL, Institute of Clinical Trials and Methodology, London, UK; 6 Data Science and Digital Innovations (DSDI), Global Statistical and Data Sciences (GSDS), BeiGene, Fulton, MD USA; 7 Center of Excellence for Statistical Innovation (CESI), Statistical Sciences & Innovation (SSI), UCB BIOSCIENCES GmbH, Alfred-Nobel-Strasse 10, 40789 Monheim, Germany; 8 Health Data Insights and Design, Global Clinical Operations, Novartis Healthcare Pvt. Ltd, Hyderabad, India; 9 Central Monitoring and Data Analytics, Global Clinical Operations, GSK, Warsaw, Poland; 10 SGH Warsaw School of Economics, Warsaw, Poland; 11 ALK, Bøge Alle 1, Hørsholm, Denmark; 12 Central Monitoring and Data Analytics, Global Clinical Operations, GSK, London, UK; 13 Amgen Inc, Thousand Oaks, CA USA

**Correction to: Therapeutic Innovation & Regulatory Science** 10.1007/s43441-023-00598-y

The author Yasha Li should have the affiliation number 3, not 2, as indicated in the published article.

